# A Protocol to Engineer Bacteriophages for Live-Cell Imaging of Bacterial Prophage Induction Inside Mammalian Cells

**DOI:** 10.1016/j.xpro.2020.100084

**Published:** 2020-08-07

**Authors:** Katie Bodner, Arin L. Melkonian, Markus W. Covert

**Affiliations:** 1 Department of Bioengineering, Stanford University, Stanford, CA 94305, USA; 2 Allen Discovery Center for Systems Modeling of Infection, Stanford University, Stanford, CA 94305, USA

## Abstract

The gut microbiome is dominated by lysogens, bacteria that carry bacterial viruses (phages). Uncovering the function of phages in the microbiome and observing interactions between phages, bacteria, and mammalian cells in real time in specific cell types are limited by the difficulty of engineering fluorescent markers into large, lysogenic phage genomes. Here, we present a method to multiplex the engineering of life-cycle reporters into lysogenic phages and how to infect macrophages with engineered lysogens to study these interactions at the single-cell level.

For complete details on the use and execution of this protocol, please refer to Bodner et al. (2020).

## Highlights

• A λ phage with a fluorescent lysis reporter is generated by yeast recombineering
• *E. coli* are infected with recombinant phage to form lysogens
• The lysis reporter is validated by agarose pad imaging and plaque assay
• Macrophages are infected with the lysogens and imaged to track prophage induction

## Graphical Abstract

**Figure:**
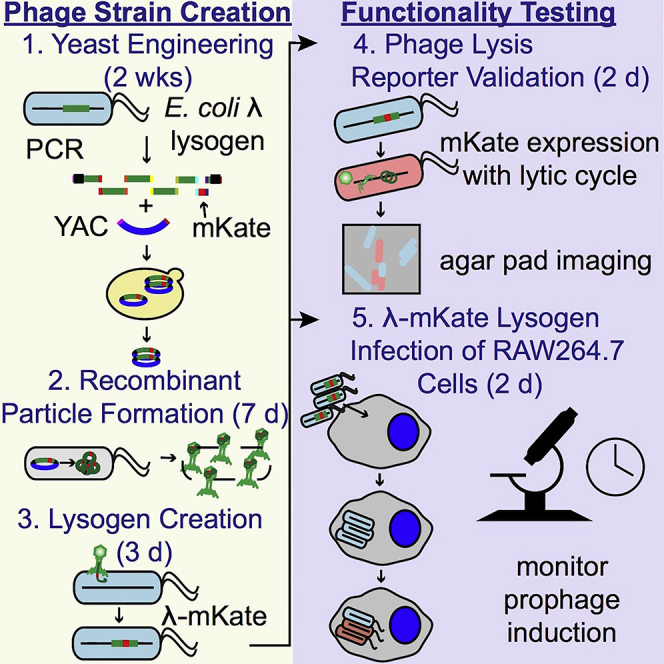

## Before You Begin

### Design Primers

**Timing: 1 day**

**Figure 1:**
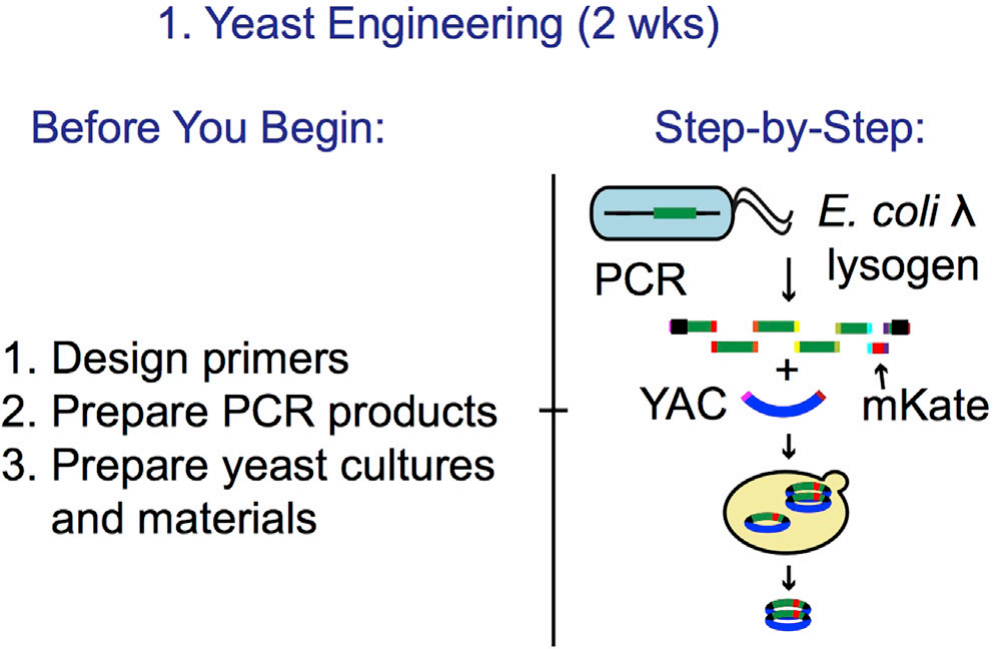
The Preparation Steps for the Yeast Engineering Protocol

Before engineering a lysogenic bacteriophage genome, we recommend generating a composite genetic map of the native bacteriophage genome in its genomic orientation inside its bacterial host. Then the phage genome from the left attachment site to the right attachment site must be inserted into a plasmid with a yeast artificial chromosome, capable of replication in yeast. Once in this orientation, fragment the genome into 10 kb even size fragments by designing PCR with primers containing homology arms to neighboring fragments. Then various mutations, selection markers or fluorescent proteins can be introduced by designing PCR products with the gene of interest flanked with homology arms to the targeted region of the phage genome. The yeast will be used to recombine all PCR fragments with homology regions and propagate the recombinant phage genome before infection into *E. coli*. In this primer design section, we demonstrate how to generate a λ phage with a fluorescent reporter for lytic transcript expression. Figure 1 describes how the Before You Begin sections including Design Primers, Prepare PCR Products and Prepare yeast cultures and materials relate to the Step-by-Step Yeast Engineering protocol.

1. Download the genbank file containing the bacteriophage genome sequence from NCBI Genome database. For this protocol, we use phage λ, which has Accession: NC_001416.1.
2. Import the phage genome sequence into a plasmid editing software. Examples include Benchling, Geneious, Snapgene, APE. In Benchling, importing a genbank file will automatically import gene annotations.
3. Annotate the phage recombination sequence, attP, (Hendrix 1983) (Figure 2).

**Figure 2:**
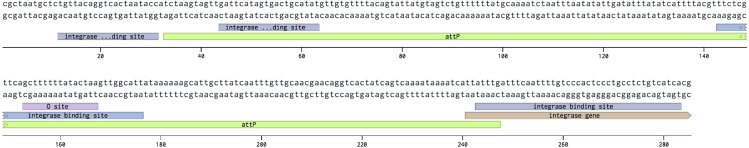
The Annotated Portion of the Genome of the Lambda Phage Containing the Recombination Site Displayed in Benchling This map shows 285 bases, corresponding to bases 27,572–27,856 of the lambda phage genome, with the core O site portion of attP annotated. Integrase binding site denotes sites where the integration enzyme, integrase binds.
4. Download the phage’s host bacterial genome in genbank format from an appropriate repository. For *E. coli* BW25113 this is: European Nucleotide Archive CP009273.1.
5. Annotate the location of the bacteriophage integration site in the bacterial genome. For phage λ, this is attB (Campbell 1992) (Figure 3).

**Figure 3:**
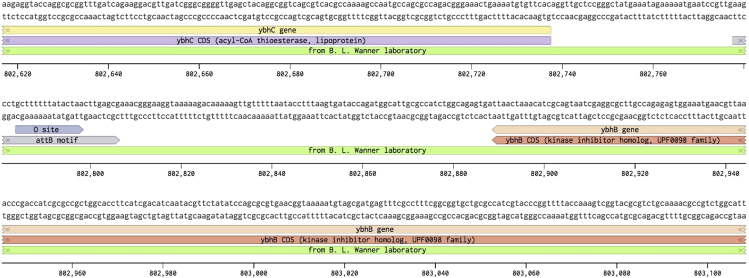
The Annotated Genome of *E. coli* BW25113 Displayed in Benchling This map shows from bases 802617 to 803108 with the core O site portion of the attB annotated along with the minimal attB motif.
6. Make a composite map of the bacteriophage genome integrated into the bacterial genome. The attL site is derived from Genbank M12458.1 and the attR site from Genbank M12459. See Figure 4.

**Figure 4:**
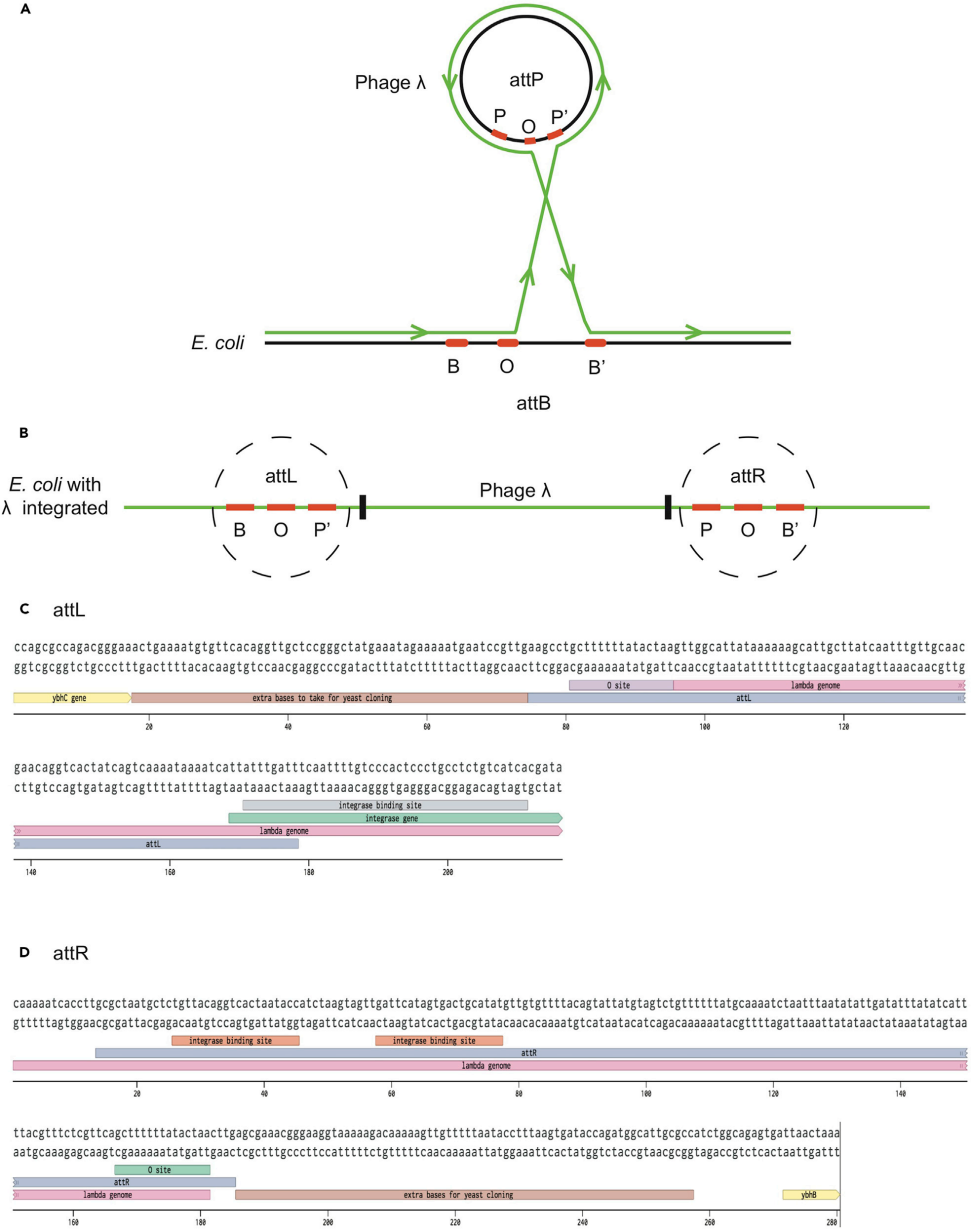
Composite Map of Phage Lambda Integrated into *E. coli* Genome (A) The process of recombination of phage attP with *E. coli* attB shown in green. (B) *E. coli* genome with lambda phage integrated and attL and attR recombined sites. (C) Sequence of the *E. coli* attL site with core O site annotated. Extra bases to take for yeast cloning indicates bases surrounding the attachment site that are included in the phage genome in the YAC construct. Integrase binding sites denote sites where the integrase enzyme binds. (D) Sequence of the attR site.

***Alternatives:*** If a composite genome sequence of the bacteriophage integrated into the bacterial genome already exists, start at the next step.

**Figure 5:**
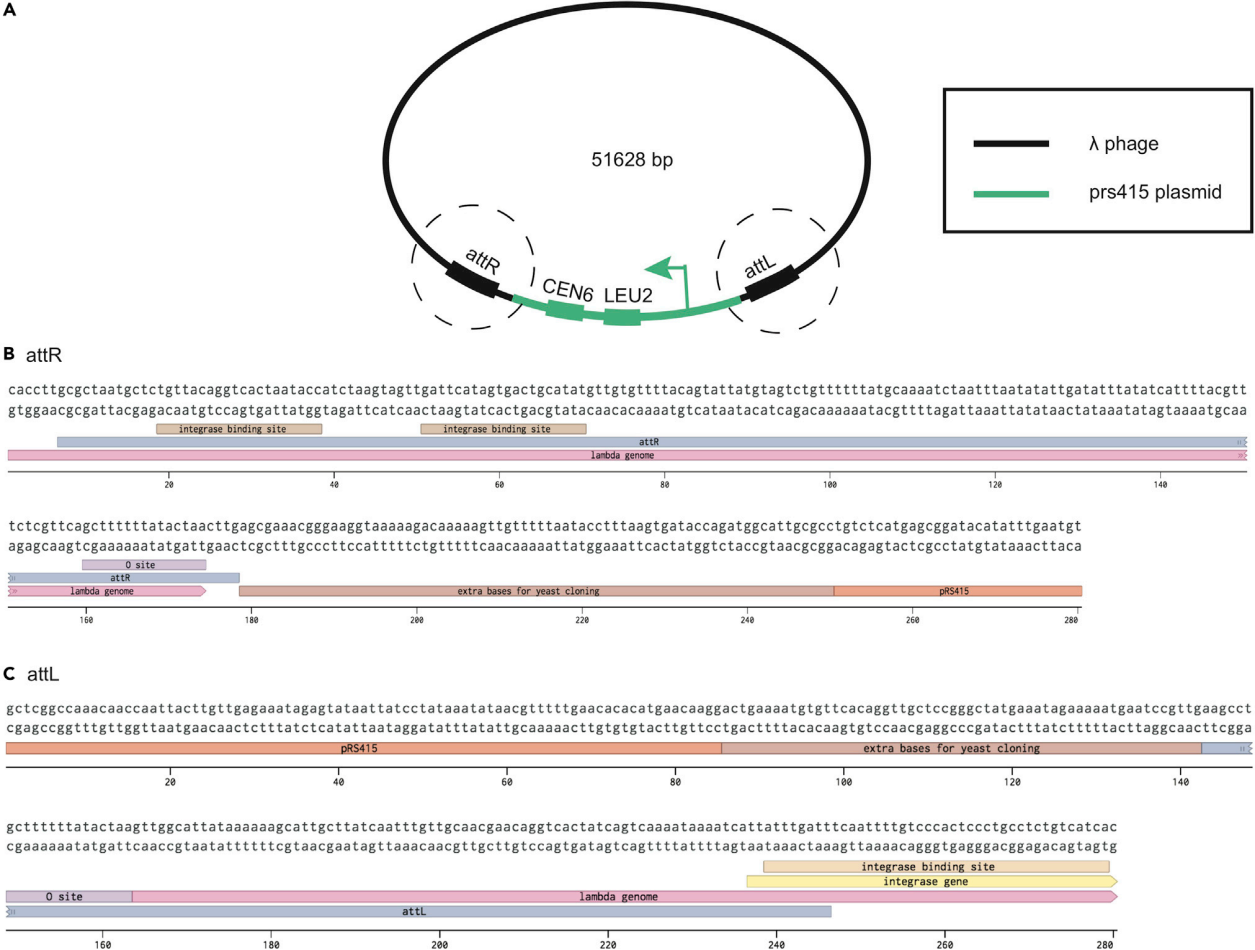
The Design of the Lambda Phage Genome within the Yeast Centromere Plasmid, pRS415 (A) The full lambda phage genome in genomic orientation from attL to attR is inserted in pRS415 after the promoter for the LEU2 gene. (B) The lambda phage genome through attR, with 72 bp downstream in the *E. coli* genome is inserted right before the *S. cerevisiae* centromere sequence. (C) The lambda phage genome from attL, including 57 bp upstream, is inserted after the LEU2 promoter.

7. Make a composite map of the λ phage genome from attL site to attR site in a plasmid containing a yeast artificial chromosome (YAC), pRS415 (Genbank U03449.1). The pRS415 plasmid is used from bases 5,351 to 2,300, including the CEN6 centromere, LEU2 coding sequence and promoter. After the LEU2 promoter, the λ phage genome in *E. coli* genomic orientation is inserted, starting 57 bp upstream of the attL site and going through 72 bp downstream of the attR site. This design choice to take extra bases padding the att sites was to allow for the prophage to properly excise from the YAC construct in later stages of cloning. See Figure 5.

***Alternatives:*** An alternative vector containing a yeast centromere sequence along with expression of a dropout gene such as Leucine, Histidine or Uracil may be used.

8. Make modifications to the phage genome to include fluorescent proteins and selection markers.
a. To generate a lysis reporter in the late lysis operon, we insert a fluorescent protein at the end of the late lysis operon, driven by the phage pR′ promoter, such that when the lysis transcript is expressed, a fluorescent protein will also be expressed. We insert the fluorescent protein, mKate2 (Shcherbo et al., 2009), along with its own synthetic ribosomal binding sequence (RBS), B0034 (http://parts.igem.org/Part:BBa_B0034) in between *ORF-*401 and *ORF*-314 at base 20,904. This location was chosen because these genes are considered to be non-essential and are also lowly expressed during lysis (Liu et al., 2013). Furthermore, the original isolate of λ (λ-UR) had one ORF covering this region called STF, short tail fiber, but the lab variant that we use, λ PaPa, has a frameshift mutation such that 401 and 314 are separate orfs, (Hendrix and Duda, 1992).

***Alternatives:*** Depending on the motivating scientific question or how well characterized the phage genome is, fluorescent proteins can also be fused to structural genes, such as the decorative capsid protein, gpD, along with a linker. See Trinh et al., 2017. If a fusion protein is generated with an abundant capsid protein, such as gpE for λ phage, then this fusion may limit phage function. For a given phage of interest, tagging a capsid protein will require characterization of capsid architecture and the abundance of each protein in the capsid. Depending on how many genes or the length of the genes to insert, non-essential genes can be removed in the phage engineering process to conserve the genome size within reasonable packaging limits. For λ phage, the genome should not be more than 5% larger than the native size (Nurmemmedov et al., 2012). We have previously removed *EA59* and *EA31* within the non-essential B2 region and *bor*, a gene used for *E. coli* serum resistance (Liu et al., 2013).

9. Include a selection marker to be used to select lysogens with the engineered phenotype. We replaced the gene, *bor*, from bases 46,440 to 48,298 with the antibiotic resistance cassette for kanamycin. We already had a λ phage variant with this insertion in place that we could use as a PCR template. (Gift from the Lanying Zeng Lab (TAMU), Trinh et al., 2017).
10. Design sets of PCR primers to amplify the WT bacteriophage genome into roughly five equal sized fragments (∼10 kb each) with 30–60 bp overlap between fragments. Design at least 3 sets of pairs for each PCR, if working with a phage that has not previously been engineered with this protocol. Since these primers will be used in genomic DNA PCR, make the primer anneal length 30 bp to prevent non-specific annealing. Try to keep the fragment size less than 10 kb for a robust PCR. See Figure 6, pink primers.

**Figure 6:**
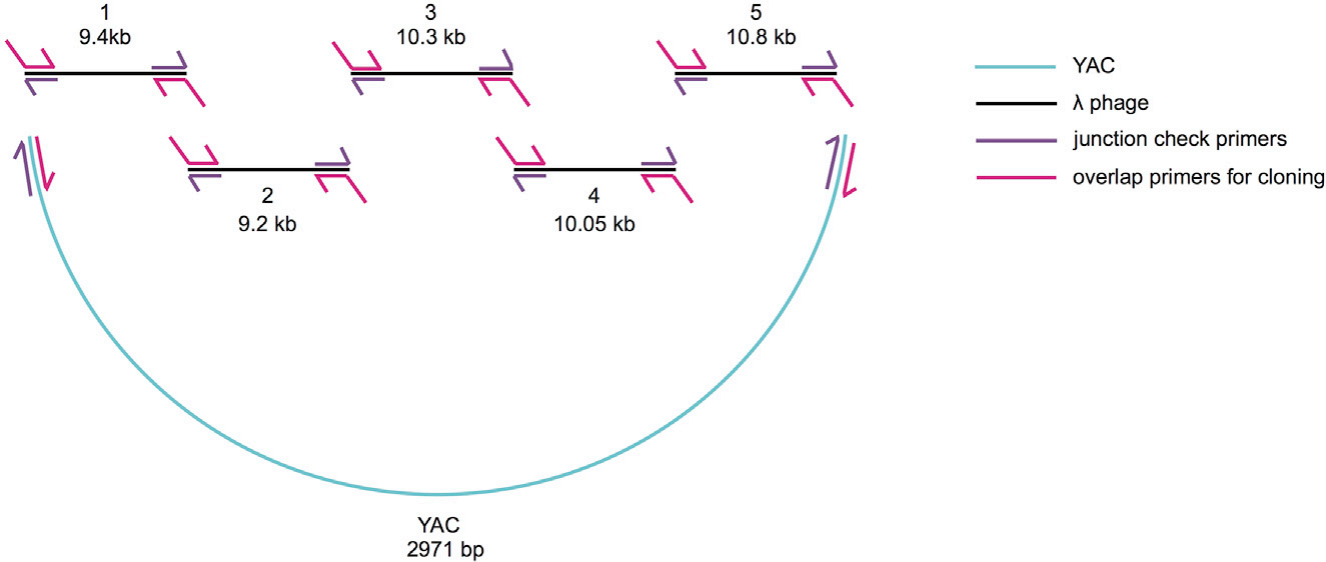
The Design of the Phage Genome in Fragments with Cloning and Validation Primers Annotated Fragment 2 contains the gene, *cI*, which can be modified to make temperature sensitive variants of the phage such as *cI857*. Fragment 3 contains the gene, *bor*, which we can replace with a kanamycin resistance cassette. Fragment 5 contains *ORF-401* and -314.
11. Decide how to break up fragments to insert the protein, mKate2. Since *ORF*-401 and -314 are in fragment 5, we redesigned fragment 5 to end right before the start of the mKate2. The mKate2 gene is amplified from a plasmid containing the mKate2 with added homology arms and is now called fragment 6. The rest of the original fragment 5 is now fragment 7. See Figure 7 for the location of the two modifications (insertion of kanamycin resistance and RBS-mKate2) in the context of the late lysis operon. See Figure 8 for the nucleotide sequences of RBS-mKate2 and the associated primers surrounding fragments 5, 6 and 7. Also, see Table 1 for the description of all genome fragments and Table 2 for all primers designed.

**Figure 7:**
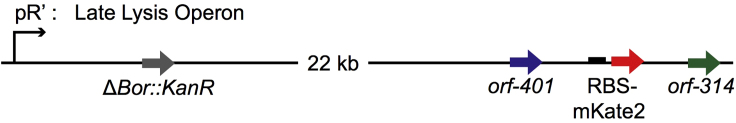
The Design of the Two Modifications to the Lambda Phage Genome in the Context of the Late Lysis Operon, Driven by the pR′ Promoter The *bor* gene is replaced with a kanamycin resistance cassette, and the RBS-mKate2 is inserted between *ORF*-401 and *ORF*-314. There are 22.248 kb between the start of the kanamycin resistance gene and the start of the RBS for mKate2.

**Figure 8:**
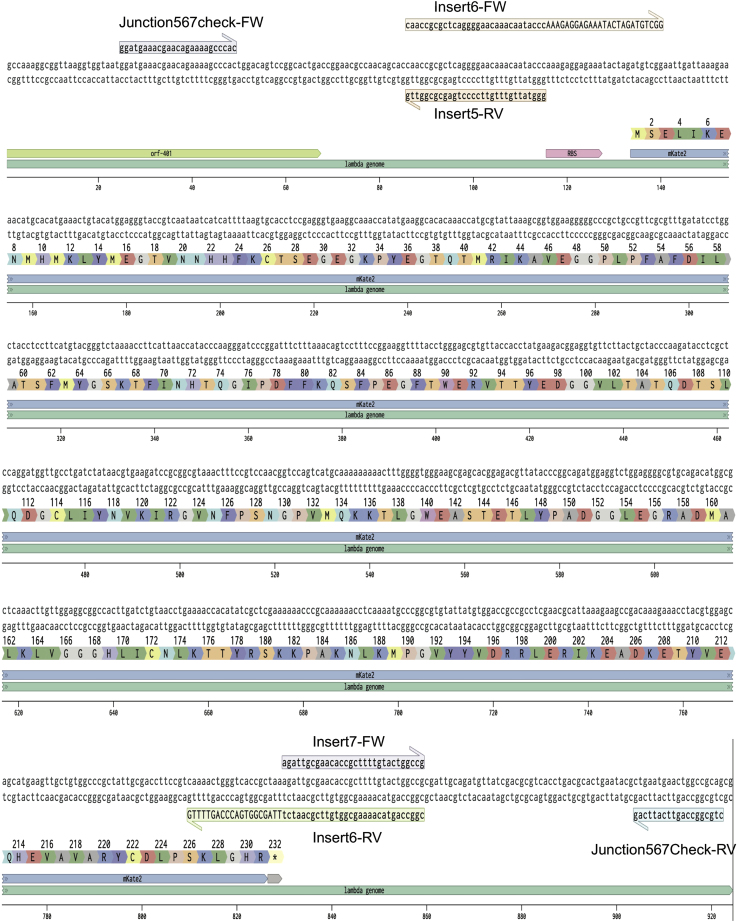
The Genome Sequences, Shown in Benchling, of the mKate2 Insertion in the Lambda Phage Genome with Primers Used for Phage Cloning and Validation Annotated

**Table 1:** The Design of the Seven Fragments Tiling the λ Phage Genome with mKate2 Inserted

Insert Number	Starting Location	Ending Location	Template	Source	Length (bp)
1	BW25113: 802721	Lambda: 37072	BW25113-Lambda lysogen gDNA	Bodner et al., 2020	9442
2	Lambda: 37008	Lambda: 46260	BW25113-Lambda or -Lambda Δ*bor::kan* (for *cI857*) lysogen gDNA	Bodner et al., 2020	9253
3	Lambda: 46149	Lambda: 7870	BW25113-Lambda Δ*bor::kan* lysogen gDNA	Gift from Lanying Zeng	9637
4	Lambda: 7791	Lambda: 17840	BW25113-Lambda lysogen gDNA	Bodner et al., 2020	10050
5	Lambda: 17761	Lambda: 20903	BW25113-Lambda lysogen gDNA	Bodner et al., 2020	3143
6	mKate-85	mKate-795	pPE005 (p15A: pL-mKate-LVA)	Bodner et al., 2020	774
7	Lambda: 20904	BW25113: 802845	BW25113-Lambda lysogen gDNA	Bodner et al., 2020	6257
YAC	pRS415: 5351	pRS415: 2300	pRS415 (Yeast Artificial Chromosome (YAC) -containing plasmid)	Gift from Erin Schwartz	2971
					

Adapted from Table S1A in Bodner et al., 2020.

**Table 2:** Primer Sets Used in This Study

Primer	Sequence	Purpose
KB135	Tataacgtttttgaacacacatgaacaaggactgaaaatgtgttcacaggttgctccggg	Amplify insert 1
KB137	gggaaagataagcgctcaataaacctgtctg	Amplify insert 1
KB253	tgatgattatcagccagcagagaattaagg	Amplify insert 2
KB258	agactgctttgatgtgcaaccgacgacgac	Amplify insert 2
KB256	gctgcgctcgatgcaaaatacacgaaggag	Amplify insert 3
KB254	tttcctcaccgatggtcagcgtgtctccac	Amplify insert 3
KB257	ctccagcccgtccctgtttgtccggactga	Amplify insert 4
KB259	atattgatactggcggctatccagtacagc	Amplify insert 4
KB255	attgcggatatcagacaggttgaaaccagc	Amplify insert 5
KB47	gggtattgtttgttcccctgagcgcggttg	Amplify insert 5
KB48	caaccgcgctcaggggaacaaacaatacccaaagaggagaaatactagatgtcgg	Amplify insert 6
KB51	cggccagtacaaaagcggtgttcgcaatctttagcggtgacccagttttg	Amplify insert 6
KB50	agattgcgaacaccgcttttgtactggccg	Amplify insert 7
KB136	acattcaaatatgtatccgctcatgagacaggcgcaatgccatctggtatcacttaaagg	Amplify insert 7
KB158	tgtctcatgagcggatacatatttgaatgt	Amplify YAC
KB159	ccttgttcatgtgtgttcaaaaacgttata	Amplify YAC
KB162	agcgcccctgtgtgttctcgttatgttgag	Validate insert YAC-1 junction
KB163	aagcatcaggtctttccttcgaaggggatc	Validate insert YAC-1 junction
KB164	aacgcgctctccactgcttaatgacattcc	Validate insert 1–2 junction
KB165	aaagttatcgctagtcagtggcctgaagag	Validate insert 1–2 junction
KB166	gcggcaattactgacatgcagatgcgtcag	Validate insert 2–3 junction
KB167	cctgattgcccgacattatcg	Validate insert 2–3 junction
KB168	accgtgattctggatacgtctgaactggtc	Validate insert 3–4 junction
KB169	aagccagagatgacaacttccgccatcatc	Validate insert 3–4 junction
KB170	tgagtttcctgctccgtctgaccgtaacag	Validate insert 4–5 junction
KB171	cactctttcgaaaactcctccagtctgctg	Validate insert 4–5 junction
KB58	ggatgaaacgaacagaaaagcccac	Validate 5–6–7 junction
KB62	ctgcggccagttcattcag	Validate 5–6–7 junction
KB172	cctttaagtgataccagatggcattgcgcc	Validate 7-YAC junction
KB173	cttgcctgtaacttacacgcgcctcgtatc	Validate 7-YAC junction
KB302	ctctggagtgcgacaggttt	binds in phage, amplifies across attR site, check for phage integration
KB303	ccttcccgtttcgctcaagt	binds in *E. coli*, amplifies across attR site , check for phage integration
a. Design sets of PCR primers to amplify across each junction between fragments to validate the phage assembly (Figure 6, purple primers). Target the primer design to include 30 bp anneal length and at least 500 bp PCR size. For small inserts such as the mKate2 insert, one set of primers can be designed to cover fragments 5–6 and 6–7 junctions. See Figure 8; Junction567Check-FW and Junction567Check-RV primers.

### Prepare PCR Products

**Timing: 2 days**

After designing PCR primers, perform phage fragment PCRs with lysogen genomic DNA templates.

12. Purify the genomic DNA used as template for fragment PCRs in Table 1 from BW25113 *E. coli* lysogenized for various λ variants including the WT and λ-Δ*bor::kan* (a gift from the lab of Lanying Zeng) using DNeasy Blood and Tissue kit (Qiagen). See the Lysogen Creation and Validation section for this protocol if you are not starting with an already lysogenized bacterial strain for the phage of interest.
13. Grow each lysogen in 3 mL of LB at 37°C, or 30°C for a temperature sensitive mutant, for 16 h from a single streaked colony.
14. Pretreat 1 mL of the overnight (grown for 16 h) culture according to DNeasy Blood and Tissue kit “Protocol: Pretreatment for Gram-Negative Bacteria.”
15. Perform the rest of the protocol starting with step 2 of “Purification of Total DNA from Animal Tissues (Spin-Column Protocol)” with the following modifications: The RNase step is performed as in step 2. In steps 4–6, it is important to replace the collection tube after each centrifuge step. DNA is eluted twice in a 100 μL volume. Expected yields are ∼3–5 μg.
16. Miniprep the pRS415 plasmid to be used as a PCR template.
17. Set up a PCR for all genome fragments and the YAC, according to Table 3, using the recipe in Table 4, with Phusion HF Polymerase, using the thermocycler settings in Table 5. Each PCR can be set up in triplicate and concentrated during purification. Yields of PCR products after purification must be at least 1 μg total to be used for yeast engineering. If the PCR reactions fail or give low yields, See Troubleshooting: Problem 1.

**Table 3:** PCR Reactions for Lambda and YAC Fragments

Fragment	Template	Forward Primer	Reverse Primers	Melting Temperature	Expected Size / Extension Time
1	BW25113/lambda WT (gDNA, 50 ng)	KB135	KB137	72°C	9,442 bp/5 min
2	BW25113/lambda WT (gDNA, 50 ng)	KB253	KB258	72°C	9,253 bp/5 min
3	BW25113/lambda Δ*bor::kan* gDNA, 50 ng)	KB256	KB254	72°C	9,637 bp/5 min
4	BW25113/lambda WT (gDNA, 50 ng)	KB257	KB259	72°C	10,050 bp/5 min
5	BW25113/lambda WT (gDNA, 50 ng)	KB255	KB47	72°C	3,143 bp/1 min, 35 s
6	Plasmid containing mKate2 (pPE005), 10 ng	KB48	KB51	64°C	774 bp/30 s
7	BW25113/lambda WT (gDNA, 50 ng)	KB50	KB136	72°C	6,257 bp/3 min, 15 s
YAC	pRS415, 10 ng	KB158	KB159	68°C	2,971 bp/ 1 min

**Table 4:** PCR Recipe for Phage Fragment PCRs

Reagent	Final Concentration (mM or μM)	Volume (μL)
Template DNA	50 ng gDNA or 10 ng plasmid DNA	Variable
Forward Primer (10 μM)	0.5 μM	2.5
Reverse Primer (10 μM)	0.5 μM	2.5
dNTPs (10 mM)	0.2 mM	1
Phusion HF Polymerase	1 unit / 50 μL PCR	0.5
5× Phusion HF or GC Buffer	1×	10
DMSO	up to 5%	up to 2.5
ddH_2_O	n/a	to 50
Total	n/a	50

**Table 5:** Thermocycler PCR Protocol for Phusion HF Polymerase

PCR Cycling Conditions			
Steps	Temperature	Time	Cycles
Initial Denaturation	98°C	2 min	1
Denaturation	98°C	10 s	40 cycles
Annealing	Melting Temperature	30 s	
Extension	72°C	15 s/kb for plasmid DNA or 30 s/kb for genomic DNA	
Final Extension	72°C	5 min	1
Hold	4°C	Forever
18. After PCR, treat the YAC fragment with the restriction enzyme, DpnI (NEB) for at least 1 h to reduce yeast genomic DNA background. Add 1 μL of enzyme directly to the PCR reaction and incubate at 37°C.

***Note:*** We have tried performing DpnI treatment for 16 h overnight to bring reaction to saturation.

19. Purify all PCR products with the QIAquick PCR purification kit (Qiagen) prior to transformation, and elute in 15 μL H_2_O to concentrate as highly as possible.

### Prepare Yeast Cultures and Materials

**Timing: 2 days**

This section details preparation of yeast growth medium, materials for yeast transformation and validation and yeast growth from glycerol stock.

20. Prepare yeast dropout medium, SD-LEU. Mix 13.35g of Minimal SD Base (Clontech) with 0.325g of -His/-Leu/-Ura DO Supplement (Clontech) in 0.5L water and autoclave at 121°C for 15 min. After medium is cool, add 20 μg/mL L-Histidine (Sigma) and 20 μg/mL Uracil (Sigma). Prepare another batch of SD-LEU (0.5L) to pour SD-LEU plates with additional 7.5 g of Bacto Agar (Thermo Fisher Scientific) added.
21. Prepare yeast growth medium, YPD broth. Add 50 g of YPD Broth (Fisher (RPI)) per 1 L of distilled water. Autoclave at 121°C for 15 min. Also, prepare another batch to pour YPD plates with 15 g of Bacto Agar added per 1 L of YPD broth.
22. Prepare a fresh solution of 50 % w/v PEG3350 (Sigma-Aldrich) in distilled water. Simultaneously stir and apply mild heat to PEG solution until fully dissolved and clear (may take ∼2–3 h).
23. Prepare 20 mM NaOH fresh on the day of yeast colony PCR.
24. Streak *S. cerevisiae* BY4741 from glycerol stock onto YPD plates and grow at 30°C for 2–3 days.

***Note:*** The yeast colonies remain stable on the plate for approximately 1 month, if kept at 4°C.

### Prepare Bacterial Culture Materials

**Timing: 2 h**

This section details *E. coli* growth media and plates, antibiotic conditions and preparation of agarose pads for live-cell imaging of *E. coli*.

25. Prepare bacterial growth medias and agar plates.
a. Unless indicated otherwise, all *E. coli* cell lines are grown with aeration in Miller’s LB Broth at 37°C.
b. For LB Broth, dissolve 25 grams of Miller’s LB Broth in 1 liter water, and autoclave for 20 min at 121°C.
c. For LB agar plates, add 37 g of Miller’s LB Agar to 1 liter water, and autoclave for 20 min at 121°C.

***Note:*** Wait for media to cool before adding any antibiotics.

d. After LB/agar media has cooled, pour ∼20 mL per 10 cm plate.
26. Unless indicated otherwise, antibiotics are applied at: 100 μg/mL carbenicillin (Fisher), 20 μg/mL kanamycin (Fisher) for use with genomic integrations or 50 μg/mL kanamycin for use with kanamycin resistance plasmids, 20 μg/mL chloramphenicol (Sigma-Aldrich) and 10 μg/mL gentamicin (Thermo Fisher Scientific).
27. Prepare agarose pad materials 1 h before performing bacterial imaging:
a. Make 1% agarose (Low-EEO/Multi-Purpose/Molecular Biology Grade, Fisher Scientific) solution in 0.4% glucose (22 mM) + M9 minimal medium (1× M9 salts (BD), 2mM MgSO_4_ (Sigma-Aldrich), 100 mM CaCl_2_ (EMD)).

**Table:** 

Reagent	Final Concentration	Amount
20% Glucose	22 mM	0.4 mL
5× M9 salts	1×	4 mL
1 M MgSO_4_	2 mM	40 μL
1 M CaCl_2_	100 mM	2 mL
agarose	1%	0.2 g
ddH_2_O	n/a	13.56 mL

b. Heat in the microwave until the agarose fully dissolves.
c. Prepare agarose pads according to the Skinner et al., 2013 protocol.
i. Pour the M9 solution onto a glass slide cast.
ii. After the gel has cooled for 20 min, use a biopsy punch to cut uniform circles pads.
iii. Transfer the pads onto a clean glass slide.
28. Prepare bacterial cultures prior to all imaging experiments
a. Streak out from glycerol stocks the lysogenic strain and non-lysogenic strain onto LB agar plates with the appropriate antibiotics.

***Note:*** Agar plates with lysogens should be stored in the dark at 4°C and used within 1 week of streaking for imaging experiments for maximal consistency.

### Prepare Phage Materials

**Timing: 2 days**

This section details *E. coli* growth medium and phage storage medium necessary for the Recombinant Phage Particle Formation and Lysogen Creation and Validation protocols.

29. Prepare TB medium for growth of *E. coli* in lysogen creation and plaque assay protocols.
a. Mix 10 g/L Bacto tryptone (Fisher Scientific) and 5 g/L NaCl (Fisher Scientific) in distilled water and autoclave.
b. Also, prepare TB agar plates by creating the same mixture as above, with 15 g/L of Bacto Agar added and autoclave. Pour 20 mL per plate. TB agar plates should be less than 1 week old and ideally freshly made for plaque assays.
30. Prepare SM medium for storing phage stocks. Mix 100 mM NaCl (Fisher Scientific), 8 mM MgSO_4_∗7H_2_0 (Sigma-Aldrich), 50 mM Tris pH=7.5 (Life Technologies)) and 0.01% w/v gelatin from porcine skin, Type A (Sigma) in distilled water and filter sterilize through a 0.2 μm filter.

**Table:** 

Reagent	Final Concentration	Amount
NaCl	100 mM	5.8 g
MgSO_4_∗7H_2_0	8 mM	2 g
1M Tris pH=7.5	50 mM	50 mL
2% w/v gelatin from porcine skin, Type A	0.01%	5 mL
ddH_2_O	n/a	up to 1,000 mL

### Prepare Mammalian Cells

**Timing: 7 days**

RAW264.7 cells are used as a mammalian macrophage-like cell line. The RAW264.7-H2B-miRFP670 cells have a nuclear marker for use in live-cell imaging. See Bodner et al., 2020 for details on cell line creation. This section details general cell culture protocols for maintaining RAW264.7 cells, which will be infected with *E. coli* lysogens in the section: Bacterial Lysogen Infection of RAW264.7 Cells.

31. Prepare general mammalian cell culture media. The RAW264.7-H2B-miRFP670 (male) cells are cultured in DMEM (ThermoFisher Scientific, 11965-118) with 10% FBS (Omega Scientific), 2 mM L-glutamine (Life Technologies), and 1× Penicillin/Streptomycin (Life Technologies) at 37°C, 5% CO_2_. These cells are derived from RAW264.7 (RRID:CVCL_0493) cells from ATCC.

**Table:** 

Reagent	Final Concentration	Amount
DMEM	n/a	440 mL
FBS	10%	50 mL
200 mM L-glutamine	2 mM	5 mL
100× Penicillin/Streptomycin	1×	5 mL

32. Prepare imaging media (IM). Mix FluoroBrite™ DMEM (Life Technologies) with 10 mM HEPES pH=7.0 (Sigma), 1% FBS and 2 mM L-glutamine.

**Table:** 

Reagent	Final Concentration	Amount
FluoroBrite™ DMEM	n/a	440 mL
FBS	1%	5 mL
L-glutamine	2 mM	5 mL
1 M HEPES, pH=7.0	10 mM	50 mL

***Note:*** Make another batch with 10 μg/mL gentamicin (Thermo Fisher, 10687). Gentamicin is used during bacterial infection of the RAW264.7 cells to prevent bacterial overgrowth in the medium.

***Note:*** Fluorobrite medium supplemented with HEPES is stable for about 4 weeks. Periodically check that the pH of the medium has not risen above 7.8 for optimal cell health.

33. Prepare RAW264.7 cells for infection.
a. Day 1: Thaw RAW264.7-H2B-miRFP670 cells from liquid nitrogen stocks and plate onto 10 cm tissue culture-treated plates in 10 mL of supplemented DMEM.

**CRITICAL:** Before cells are confluent, check for good cell adherence post-thaw. If dead cells are floating in the media, perform media changes prior to splitting.

b. Day 4 (or when cells have reached 90% confluence): Remove media and apply 3 mL of accutase onto the cells and incubate for 15 min at 37°C. Split cells 1:5 onto another 10 cm plate.

***Note:*** The RAW264.7-H2B-mIRFP670 cells were engineered with a lentivirus and also contain a hygromycin resistance cassette. For long-term maintenance of this cell line, supplement the media with 250 μg/mL hygromycin B to prevent genetic drift.

c. Continue to sub-culture cells, maintaining at less than 90% confluence, until ready for bacterial infection. See section: Bacterial Lysogen Infection of RAW264.7 Cells

## Key Resources Table

**Table:** 

REAGENT or RESOURCE	SOURCE	IDENTIFIER
**Bacterial and Virus Strains**		

*Escherichia coli* strain MG1655	Coli Genetic Stock Center	CGSC#7740
*Escherichia coli* strain BW25113	Baba et al., 2006	N/A
*Escherichia coli* strain MG1655/PE003	Bodner et al., 2020	N/A
*Escherichia coli* strain BW25113/PE003	Bodner et al., 2020	N/A
*Escherichia coli* strain MG1655 attB::[λ-mKate *cI* (WT)]/ PE003	Bodner et al., 2020	N/A
*Escherichia coli* strain BW25113 attB::[λ-mKate *cI* (WT)]/ PE003	Bodner et al., 2020	N/A
*Escherichia coli* strain MG1655/pUC19	Bodner et al., 2020	N/A
Bacteriophage λ	ATCC	Cat#23724-B2
Bacteriophage λ Δ*bor::kan cI*857	Gift from the Lanying Zeng Lab (TAMU)	N/A
Bacteriophage λ-mKate: *cI* (WT) Δ*bor::kan*, RBS-mKate2, Δ[*ea31,ea59*]::PGK-mClover-bgh terminator	Bodner et al., 2020	N/A
*E. coli* MegaX DH10B T1R Electrocomp Cells	Thermo Fisher Scientific	Cat# C6400-03
*E. coli* MegaX DH10B attB::[λ-mKate *cI* (WT)	Bodner et al., 2020	N/A

**Chemicals, Peptides, and Recombinant Proteins**		

FluoroBrite™ DMEM	Life Technologies	Cat#A1896702
DMEM	ThermoFisher Scientific	Cat#11965-118
FBS	Omega Scientific	Cat#FB-12
L-glutamine	Life Technologies	Cat#25030081
Penicillin - Streptomycin (100×)	Life Technologies	Cat#15140-122
HEPES pH=7.0	Sigma	Cat#H0887-100ML
Fibronectin	Sigma-Aldrich	Cat#F0895-5MG
Hygromycin B	Thermo Fisher Scientific	Cat#10687010
Gentamicin	Thermo Fisher Scientific	Cat#15710064
Norfloxacin	Sigma-Aldrich	Cat#N9890-5G
gelatin from porcine skin, Type A	Sigma-Aldrich	Cat#G1890-100G; Cas#9000-70-8
Accutase **®**	Sigma-Aldrich	Cat#A6964-100ML
Phusion High-Fidelity DNA Polymerase	New England Biolabs	Cat# M0530S
GoTaq® Green Mastermix	Promega	Cat#M7122
RNase A	Qiagen	Cat#19101
DpnI	New England Biolabs	Cat#R0176S
Difco™ M9 Minimal Salts, 5×	BD	Cat#248510
Minimal SD Base	Takara Bio	Cat#630411
DO Supplement -His/-Leu/-Ura	Takara Bio	Cat#630425
L-Histidine	Sigma	Cat#H8000-25G
Uracil	Sigma	Cat#U0750-25G
YPD Broth	Fisher Scientific (RPI)	Cat#50-489-171
PEG3350 (Polyethylene glycol average M_n_ 3,350, powder)	Sigma-Aldrich	Cat#202444-250G
Carbenicillin (Disodium Salt)	Fisher Scientific	Cat#BP26485
Kanamycin Monosulfate	Fisher Scientific	Cat#K00475G
Chloramphenicol	Sigma-Aldrich	Cat#C0378-25G
Maltose Monohydrate	EMD Millipore	Cat#MX0160-1
Sheared Salmon Sperm DNA	Invitrogen	Cat# AM9680
Sodium Hydroxide	Sigma	Cat#S5881-500G
Luria Agar Granulated [Miller’s LB Agar]	RPI	• Cat#L24022-1000
LB Broth (Miller) Mix	Genesee Scientific	Cat#11-118
BD Bacto™ Dehydrated Agar	Thermo Fisher Scientific	Cat#DF0140-01-0
Agarose (Low-EEO/Multi-Purpose/Molecular Biology Grade)	Fisher Scientific	Cat#BP160-100
D-(+)-Glucose	Sigma	Cat#G8270
Magnesium Sulfate	Sigma-Aldrich	Cat#M2643-500G
Calcium Chloride Dihydrate	EMD Millipore	Cat#1023820250
BD Bacto™ tryptone	FPI Fisher Scientific	Cat#50-213-717
Sodium Chloride	Fisher Scientific	Cat#S271-1
UltraPure™ 1 M Tris-HCl Buffer pH=7.5	Life Technologies	Cat# 15567-027

**Critical Commercial Assays**		

DNeasy Blood and Tissue Kit	Qiagen	Cat#69504
YeaStar™ Genomic DNA Kit	Zymo Research	Cat# D2002
QIAquick PCR Purification Kit	Qiagen	Cat# 28106

**Experimental Models: Cell Lines**		

Mouse: RAW264.7 cells (male)	ATCC	Cat#TIB-71; RRID:CVCL_0493
Mouse: RAW264.7 cells (male), pGK-HygroR,pGK-H2B-miRFP670	Bodner et al., 2020	N/A

**Experimental Models: Organisms/Strains**		

*Saccharomyces cerevisiae strain BY4741, MATa his3*Δ*1 leu2*Δ *met15*Δ *ura3*Δ	Gift from the Onn Brandman Lab (Stanford)	N/A
*Saccharomyces cerevisiae strain BY4741, MATa his3*Δ*1 leu2*Δ *met15*Δ *ura3*Δ / pRS415-λ-mKate	Bodner et al., 2020	N/A

**Oligonucleotides**		

Primers for recombinant phage construction and validation and Keio strain validation, see Table 2	Bodner et al., 2020	N/A

**Recombinant DNA**		

Plasmid PE003: SC101 (CmR) - J23119-B0030-mCerulean3 (constitutive bacterial mCerulean expression plasmid)	Bodner et al., 2020	N/A
Plasmid pRS415 (yeast centromere plasmid for expression of LEU2, with bacterial origin of replication), (AmpR)- LEU2, CEN/ARS (*S. cerevisiae* centromere CEN6 fused to replicating sequence*)*	Gift from Erin Schwartz (Stanford)	N/A
Plasmid pUC19	Thermo Fisher Scientific	Cat#SD0061
Plasmid PE005: p15A: pL-mKate-LVA	Bodner et al., 2020	N/A

**Software and Algorithms**		

Micro-Manager 2.0	Edelstein et al., 2014	https://micro-manager.org/wiki/Version_2.0
Benchling	Benchling [Biology Software]. (2020). Retrieved from https://benchling.com.	https://benchling.com/

**Other**		

Nunc™ Microwell™ 96-Well Optical-Bottom Plates with Coverglass Base	Thermo Fisher Scientific	Cat#164588
AeraSeal™ film	Sigma-Aldrich	Cat#A9224
Fisherbrand™ Electroporation Cuvettes Plus	Fisher Scientific	Cat# FB101
Thermo Scientific™ Nunc™ Lab-Tek™ Chambered Coverglass	Fisher Scientific	Cat#12-565-472
CELLSTAR® Tissue Culture Plates, Greiner Bio-One	VWR	Cat#655161
Petri dish 100 × 15 mm	Fisher Scientific	Cat# FB0875713
Eclipse Ti fluorescence microscope	Nikon	Ti-E

## Step-By-Step Method Details

### Yeast Engineering

**Timing: 7–11 days**

This protocol describes how to recombine large fragments of phage genomic DNA in yeast and how to extract and validate the genomic DNA containing the YAC- λ construct. Steps include yeast culture, genome assembly by transformation of phage DNA into competent yeast cells, genomic DNA extraction, and transformant validation by PCR. Depending on the desired modifications to the phage genome, simpler phage engineering protocols could be substituted such as recombineering in bacteria instead of yeast (see Thomason et al., 2009). We chose the yeast recombineering method as it is easier to multiplex multiple modifications to the phage genome simultaneously, does not need a separate selection marker per modification generated and requires screening fewer colonies.

1. Pick single colonies of *S. cerevisiae* BY4741 and inoculate in 3 mL YPD broth a day prior to yeast recombination protocol and grow at 30°C for 16 h.
2. Dilute the overnight (grown for 16 h) cultures 1:10 into 30 mL of YPD broth and incubate at 30°C for 4 h.
3. Centrifuge cells at 1,000 × *g* for 5 min at 24°C.
4. Wash with 25 mL water, and centrifuge as step 3.
5. Wash with 1 mL of 100 mM lithium acetate (LiAc), and centrifuge as step 3.
6. Perform the final resuspension in 240 μL of 100 mM LiAc.

**CRITICAL:** After resuspension in LiAc, competent cells are promptly used for transformation and not stored.

7. Mix all PCR products (phage genome fragments and YAC) in an eppendorf tube up to a total volume of 50 μL, including H_2_O.
a. Add approximately 1 μg of DNA per fragment and 300 ng of YAC.

***Note:*** The DNA should be concentrated during the PCR purification step to reach ∼1,000 ng/μL so that only 1 μL of each fragment is added.

b. In addition, add approximately 1 μg of pRS415 plasmid DNA to 50 μL of water as a positive control and 300ng of the YAC vector to 50 μL of water as a negative transformation control.

***Note:*** The original yeast recombineering protocol (Ando et al., 2015), recommends 400 ng DNA/fragment; however, we have found the protocol to be more robust with 1 μg of DNA/fragment and at least 3× molar excess for fragments less than 1 kb.

8. Boil 2 mg/mL salmon sperm DNA (Invitrogen) at 95°C for 5 min.
9. Make three transformation mixtures, each with 50 μL yeast competent cells, 240 μL 50% PEG3350 (Sigma-Aldrich), 36 μL 1 M LiAc and 25 μL boiled salmon sperm DNA.
10. Combine each of the DNA mixtures (reaction mixture, negative and positive controls) with a transformation mixture.
11. Vortex the mixture gently at medium speed.
12. Incubate at 30°C for 30 min in a rotating nutator.
13. Incubate the mixture at 42°C for 45 min.
14. Centrifuge at 8,000 × *g* for 30 s and resuspend in 200 μL water.
15. Plate the transformants on SD-LEU plates and incubate at 30°C for 3–5 days.
16. After colonies are visible, transformants can be screened for proper assembly. The positive control transformed with prs415 should have close to a lawn of colonies after plating the whole transformation volume. If the transformation efficiency is low, see Troubleshooting: Problem 3.
17. Pick 8 colonies and resuspend each in 30 μL of H_2_O.
18. Mix 20 μL of colony suspensions with 100 μL of freshly made 20 mM NaOH and incubate at 95°C for 10 min.

***Note:*** This step can be reduced to 5 min of incubation.

**CRITICAL:** Save the remainder of the colony suspension for liquid growth.

19. Vortex and centrifuge at 8,000 × *g* for 1 min.
20. Use 1 μL of lysate as a template in a colony PCR reaction with each of the junction check primers using GoTaq Green Mastermix (Promega). See Table 2 for primer sets, Table 6 for reaction mixture and Table 7 for thermocycler setup (see Figures S1A and S1B of Bodner et al., 2020 for example junction PCR design and gel electrophoresis)

**Table 6:** PCR Recipe for Junction Verification Colony PCR

Reagent	Final Concentration (mM or μM)	Volume (μL)
Template DNA (bacterial colony resuspension in water or yeast colony resuspension in NaOH)	n/a	1
Forward Primer (10 μM)	0.5 μM	1
Reverse Primer (10 μM)	0.5 μM	1
GoTaq Green Master Mix 2×	1×	10
ddH_2_O	n/a	7
**Total**	**n/a**	**20**

**Table 7:** Thermocycler PCR Protocol for GoTaq Polymerase

PCR Cycling Conditions			
Steps	Temperature	Time	Cycles
Initial Denaturation	95°C	5 min	1
Denaturation	95°C	30 s	35 cycles
Annealing	55°C	30 s	
Extension	72°C	1 min/kb for genomic DNA	
Final Extension	72°C	5 min	1
Hold	4°C	Forever

***Note:*** Validate the two outside junctions between the phage and the YAC and all of the new junctions introduced before proceeding to check all junctions.

21. For colonies containing all correct junctions, inoculate 10 μL of suspension in 20 mL of SD-LEU liquid medium and grow cultures at 30°C for 3–5 days (or until OD600=1.0 is reached).

**Pause Point:** Yeast cultures containing YAC- λ constructs can be glycerol stocked (15% glycerol) and stored at −80°C.

22. Extract total yeast genomic DNA (gDNA) from a volume of cells containing OD600=10 equivalent from the liquid cultures using the YeaStar™ Genomic DNA Kit (Zymo Research) using protocol I.
a. Typical yields range from 30–70 ng/μL when eluted in 30 μL volume.

**CRITICAL:** Perform PCR reactions for all junctions once more with the junction primer sets to confirm unwanted recombination did not occur during growth.

**Pause Point:** Yeast genomic DNA can be stored for several weeks at 4°C prior to bacterial electroporation.

### Recombinant Phage Particle Formation

**Timing: 7 days**

These steps describe how to generate or reboot phage particles from phage genomic DNA extracted from yeast. After the YAC- λ genome is electroporated into *E. coli*, the phage will excise, replicate, and form viral particles. The λ genome should excise from the YAC just as it would from the *E. coli* genome and viral particles will go on to infect untransformed cells, integrate, and form lysogens, which can be selected for with an antibiotic resistance gene on the phage. A plaque assay to detect spontaneous generated phage particles from the lysogen is then performed by mixing lysogens with a susceptible non-lysogen strain to generate plaques. Ultimately, plaques are propagated to form a high titer stock of liquid recombinant phage particles.

23. Mix 2.5 μL validated yeast gDNA with 25 μL of *E. coli* MegaX DH10B T1R Electrocomp Cells (Thermo Fisher Scientific) on ice. Add 25 μL of transformation mixture in a 1-mm gap electroporation cuvette (Fisher) and electroporate at 2,000 V, 25 μF, and 200 Ω.

***Note:*** Chill cuvette, eppendorf tubes and pipette tips on ice prior to electroporation.

***Alternatives:*** DH10B cells were chosen as a highly electrocompetent, λ negative and T1 phage resistant strain, along with genetic recombination deficiencies. Other electrocompetent strains that have efficiency close to 10^10^ cfu (colony forming units)/μg of pUC19 DNA may be used, but homemade electrocompetent strains likely do not have efficiency high enough for this protocol. For engineering other phages besides λ, this competent cell line must be able to be lysogenized by the phage of interest. A high efficiency protocol for generating electrocompetent cells for other bacterial cell lines of interest is necessary.

24. Mix cells with 1 mL of Recovery Medium (Thermo Fisher Scientific) and recover at 37°C for 3 h.

***Alternatives:*** This recovery medium may be replaced with a standard transformation rich recovery medium such as SOC or a media specific to another bacterial line protocol.

25. Plate onto LB agar plates supplemented with 20 μg/mL kanamycin, and incubate for 16 h at 37°C.

***Note:*** If working with a temperature sensitive mutant of *cI* such as *cI857*, then this step should be performed at 30°C.

***Note:*** Formation of kanamycin resistant lysogens suggests that the phage was able to excise from the YAC-phage construct, form particles and infect and lysogenize untransformed *E. coli*.

***Note:*** Plates may have only several colonies of lysogens. The phage-YAC construct makes up 0.4% of the total yeast genomic DNA transformed into *E. coli*.

26. Pick several colonies per lysogen, resuspend in 20μL H_2_O, and use 1 μL as template in a colony PCR reaction with primers across the attR site to validate that the attR site formed as a result of phage integration. Use primers KB302, KB303 and follow the reaction mixture in Table 6 and thermocycler settings in Table 7.

***Note:*** This colony PCR protocol assumes that the strain of bacteria can be denatured in the first 95°C step.

a. Perform another PCR to validate the lysogens formed with the recombinant phage particles rather than contaminating WT phage. Perform a Phusion PCR reaction using the insert 6 primers (KB48, KB51) across the mKate cassette and purify with the QIAquick PCR Purification Kit, according to the standard protocol, with the following modifications:
i. For optimal yield, elute DNA in a small volume, 15 μL, and incubate the product for 1 in at RT prior to centrifugation.
b. Send off PCR product directly for sequencing to confirm there was no cross-contamination with WT λ to form the lysogens.
27. Isolate recombinant λ mKate phage particles produced by the DH10B lysogens by detecting spontaneous induction in a plaque assay when mixed with a susceptible indicator strain. First, generate plating cells to be used as a susceptible indicator strain. Grow phage-susceptible MG1655/pUC19 *E. coli* for 16 h in 3 mL of LB supplemented with carbenicillin at 37°C.

***Note:*** The pUC19 provides carbenicillin resistance for the spontaneous induction assay where the donor phage strain has no antibiotic resistance. When the donor strain and the plating cells are mixed in the plaque assay, the released phages should infect the plating cells and form plaques. The donor bacterial strain will be killed by the carbenicillin on the plates. The choice of pUC19 was arbitrary. Any plasmid that has no effect on phage life cycle and has a single antibiotic resistance gene can be used in this step along with the corresponding antibiotic plates.

28. The next morning, dilute *E. coli* plating cells 100-fold into 20 mL TB + 0.2% maltose (EMD Chemicals) and culture at 37°C until OD600=0.4 (roughly 2 h).
29. Incubate cells on ice for 15 min.
30. Centrifuge cells at 3,000 × *g* for 10 min at 4°C.
31. Resuspend in cold TB + 10 mM MgSO_4_ (Sigma-Aldrich) to a final OD600=2.0.

**Pause Point:** Plating cells remain viable for use when stored at 4°C for up to 1 week.

32. Grow the lysogens from step 26 for 16 h in 3 mL LB + kanamycin. Dilute 1:100 in LB + 10mM MgSO_4_ + 0.2% glucose and grow at 37°C until mid-exponential phase.

***Note:*** If working with a temperature sensitive mutant of *cI* such as *cI857*, then this step should be performed at 30°C. Furthermore, a 15 min heat shock should be performed prior to step 33 at 42°C.

***Note:*** The glucose is added to the growth medium to repress expression of the phage receptor, LamB. This prevents uninduced bacteria from taking up released phage particles.

33. Mix 100 μL of plating cells and 100 μL of lysogen culture along with 3 mL of TB soft (0.7%) agar and plate onto TB Carb plates and incubate for 16 h at 37°C.

***Note:*** Include a plating cell only control to control for contamination.

***Note:*** The Carb allows a lawn of only the plating cells to grow, and any spontaneously produced particles should propagate as plaques.

**Figure 9:**
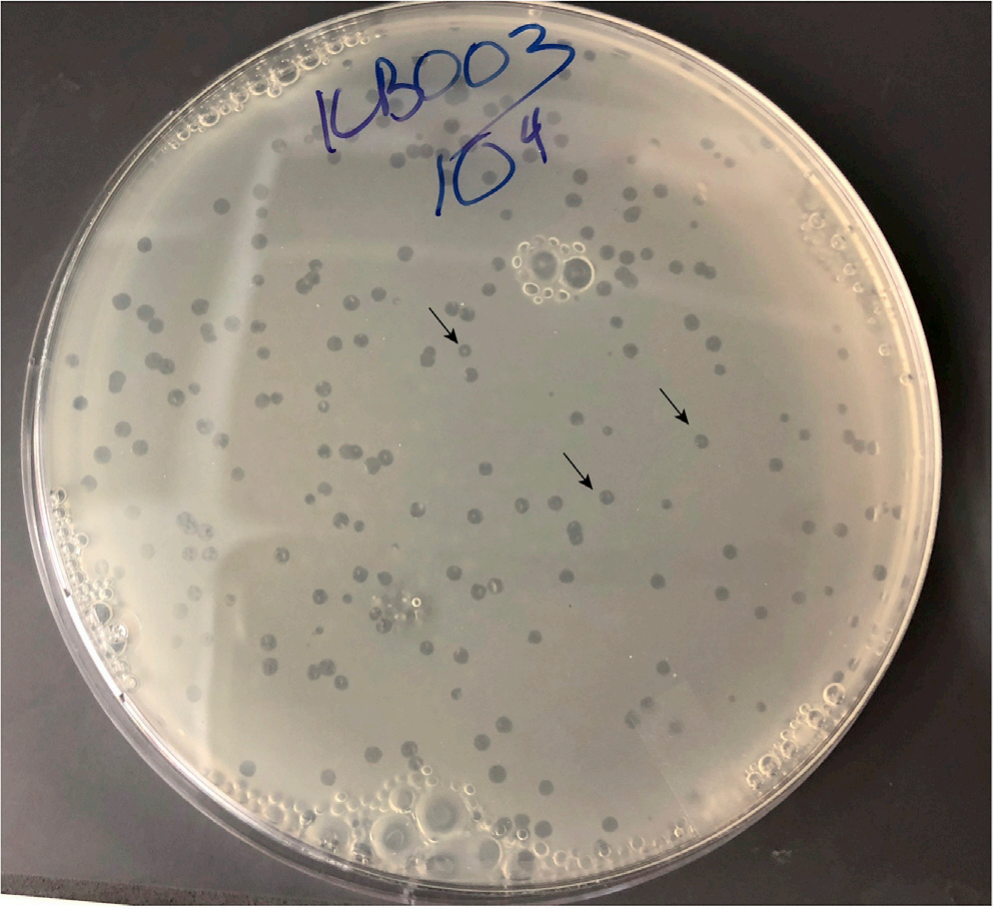
Example Plaques from λ-mKate An example TB agar plate from a titering protocol of a lawn of MG1655 *E. coli* mixed with a 10^4^ dilution of λ-mKate. Arrows indicate examples of cloudy plaques.

***Note:*** Make sure to pick the plaque by carefully skimming the “top agar”.

***Note:*** Cloudy plaques are those that have a region of growth in the center of the plaque with a zone of clearance around the center. This indicates the formation of lysogens within the plaque. If the plaque is totally clear with no growth center, this could indicate a purely lytic mutant phage.

34. Pick several “cloudy” plaques and store in 100 μL of SM+gelatin buffer at 4°C. If there are no obvious plaques, see Troubleshooting: Problem 2. See Figure 9 for an example of cloudy plaque morphology.

35. Once more, validate the phage for the presence of the mKate by PCR.

**Pause Point:** Plaques can be stored at 4°C in the dark until ready for step 36.

36. Propagate the plaque to increase phage titer by performing a plate lysis protocol. First, grow a propagation host (MG1655/pUC19) for 16 h overnight in LB.
37. The following morning, prepare propagation plates for phage. Mix 1.2% Bacto Agar (Thermo Fisher Scientific, DF0140), 0.3% glucose, 2 mM MgSO_4_ (Sigma-Aldrich), 0.075 mM CaCl_2_ (EMD), 0.004 mM FeCl_3_ (Sigma-Aldrich) and 0.01 mg/L Thiamine Hydrochloride (Sigma-Aldrich) in distilled water and autoclave. Pour plates and let solidify while performing steps 38–39.
38. After 16 h growth, dilute the bacterial propagation host culture 100-fold into 20 mL TB and culture until OD600=0.4 at 37°C.
39. Mix the resuspended plaques with 1 mL of propagation host, vortex at medium speed for 10 s and incubate at 4°C for a minimum of 15 min (up to a couple of hours), and mix 350 μL of the mixture with 3 mL of soft TB (0.7%) agar and plate onto propagation plates.
40. Incubate the plates at 37°C without inversion until plaques cover the whole plate, typically <5 h.
41. Add 5 mL of SM buffer to each plate and incubate for 2 h on a shaking platform.

**Pause Point:** SM incubation can proceed for 16 h overnight.

42. Re-collect 5 mL of buffer from the plate and add back 1 mL of fresh SM to the plate for 15 min at 4°C.
43. Combine the second SM harvest with the first, mix with 100 μL of chloroform (Sigma-Aldrich) and incubate at 24°C for 10 min.
44. Centrifuge lysates at 4,000 × *g* at 4°C for 10 min.
45. Collect the top layer (above the chloroform) in a conical tube, wrap in aluminum foil to store in the dark, and store at 4°C.

**Pause Point:** We now have our stock of phage particles that can be stored until ready to titer.

46. Determine titers by plaque assay. Mix 50 μL of plating cells with 50 μL of phage stock (in serial dilutions from 1:10^3^ to 1:10^9^).
a. Incubate plating cell/phage mixture at 37°C for 20 min.
b. Mix 100 μL of phage/cells mixture with 3 mL of soft agar (TB + 0.7% agar) and plate onto TB Agar Carb plates.
c. Incubate overnight for 16 h at 37°C.
d. Count the number of plaques on the plate that contains between 10 and 100 plaques, multiply by the dilution factor and divide by the plating volume of the phage to get the titer in PFU (plaque forming units)/mL.
e. Titers tend to be ∼10^9^-10^10^ pfu/mL after plate lysis.

**Pause Point:** We now have our stock of phage particles ready for creating lysogens.

### Lysogen Creation and Validation

**Timing: 3 days**

Once phage particles are generated, these steps cover how to infect a non-lysogenic bacterial strain to generate lysogens with the recombinant phage. After infection and lysogen creation is complete, the bacterial strain that has phage integrated within its genome must be validated by colony PCR.

47. Grow parent bacterial strains overnight (∼16 h) in LB supplemented with the appropriate antibiotic.

***Note:*** We have used both MG1655 and BW25113 *E. coli* lab strains in the λ infection protocol. These were transformed with a constitutive mCerulean3 expression plasmid (see Key Resources Table) for compatibility with downstream live-cell imaging. The mCerulean expression plasmid contains a chloramphenicol resistance cassette.

48. Dilute 100-fold into fresh TB supplemented with 0.2% maltose, and grow until early stationary phase (roughly 5 h).

***Note:*** Maltose is specific to stimulating lamB receptor expression for the lambda phage; other

bacterium/phage pairs may require different stimuli to express the phage receptor.

49. Centrifuge cells at 8,000 × *g* for 1 min at 24°C and resuspend in 1 mL of 10 mM MgSO_4_.
50. Incubate for 1 h at 30°C.
51. Mix 50 μL of cells with 50 μL phage at Multiplicity of Infection (MOI) 0.01 and incubate at 4°C for 30 min.
52. Combine the mixture of cells and phage with 900 μL of pre-warmed LB + 10 mM MgSO_4_ and incubate for 45 min at 30°C.
53. Plate 100 μL of this mixture onto LB + kanamycin plates to select for kanamycin resistant phage lysogens.
54. Incubate plates at 37°C for 16 h.

***Note:*** If working with a temperature sensitive mutant of *cI* such as *cI857*, then this step should be performed at 30°C.

55. Pick several colonies with an inoculating loop and resuspend in 20 μL of H_2_O.
56. Validate all generated lysogens by performing a colony PCR using 1 μL as template, as in step 26, with primers across the attR site to ensure phage integration.

**Pause Point:** The validated lysogens can be grown for 16 h overnight at 37°C in LB with kanamycin and glycerol stocked (15% glycerol) and stored at −80°C.

### Lysis Reporter Functional Validation

**Timing: 2 days**

The next steps demonstrate how to validate the function of the phage lysis reporter using live-cell agar pad microscopy and plaque assay. By live-cell imaging, the reporter can be validated by observing mKate fluorescence as a proxy for phage induction in the lysogen. Lysogens are stimulated with a DNA-damaging antibiotic, norfloxacin (NFX). DNA-damaging antibiotics such as norfloxacin are known to stimulate the bacterial SOS response, the main pathway responsible for prophage induction with lambda phage in *E. coli* (Matsushiro et al., 1999). Stimulated lysogens are then spotted onto an agar pad and imaged for several hours. Recombinant lysogens should also be validated by plaque assay comparison with the WT phage.

57. Validate that the recombinant phage does not have different induction behavior from the WT by performing a plaque assay for spontaneous induction. First, inoculate single colonies of lysogens for the same *E. coli* strain of recombinant λ and WT- λ in 3 mL of LB at 37°C for 16 h.
58. The next day, back-dilute the recombinant and WT lysogens in 3 mL of LB supplemented with 10mM MgSO_4_ + 0.2% glucose and grow at 37°C until mid-exponential phase (OD600=0.4).
59. Serial dilute each of the bacterial cultures in 1 mL of TB from 1:10 to 1:10^9^.
60. Mix 50 μL of each dilution with 50 μL of plating cells (see steps 27–31 in Recombinant Phage Particle Formation), and incubate at 37°C for 15 min to allow for phage DNA injection.
61. Add 100 μL of phage mixture to 3 mL of 0.7% TB soft agar and plate onto TB Agar plates supplemented with carbenicillin.
62. Incubate plates overnight (16 h) at 37°C.
63. The following day, 16 h later, count the number of plaques on the plate with between 10 and 100 plaques, and calculate the spontaneous induction frequency (PFU/CFU) for the recombinant λ and WT- λ.
64. Next validate that the phage reporter functions by performing live-cell imaging. Inoculate and grow single colonies of non-lysogenic *E.* coli and lysogenic *E. coli* strain of recombinant λ in 3 mL of LB at 37°C for 16 h.
65. The next day, dilute bacterial cultures 100-fold into two flasks each of fresh LB, and grow until mid-exponential phase (typically 2 h until OD600=0.4).
66. Add 50 ng/mL NFX to the lysogen and incubate at 37°C for 30 min. Keep the second culture unstimulated and continue to grow for 30 min.

***Alternatives:*** Depending on the phage and bacterial strains of interest, other prophage induction stimuli may be used. Other DNA-damaging antibiotics may be supplemented, such as mitomycin C, to trigger the SOS response, or UV light or heat shock for temperature sensitive mutants.

67. Concentrate each strain to OD600=0.2 and spot 1 μL onto individual agarose pads (described in Before You Begin: Prepare Bacteria Culture Materials). See Figure 10 for detailed instructions on spotting and transferring the agarose pads.

**Figure 10:**
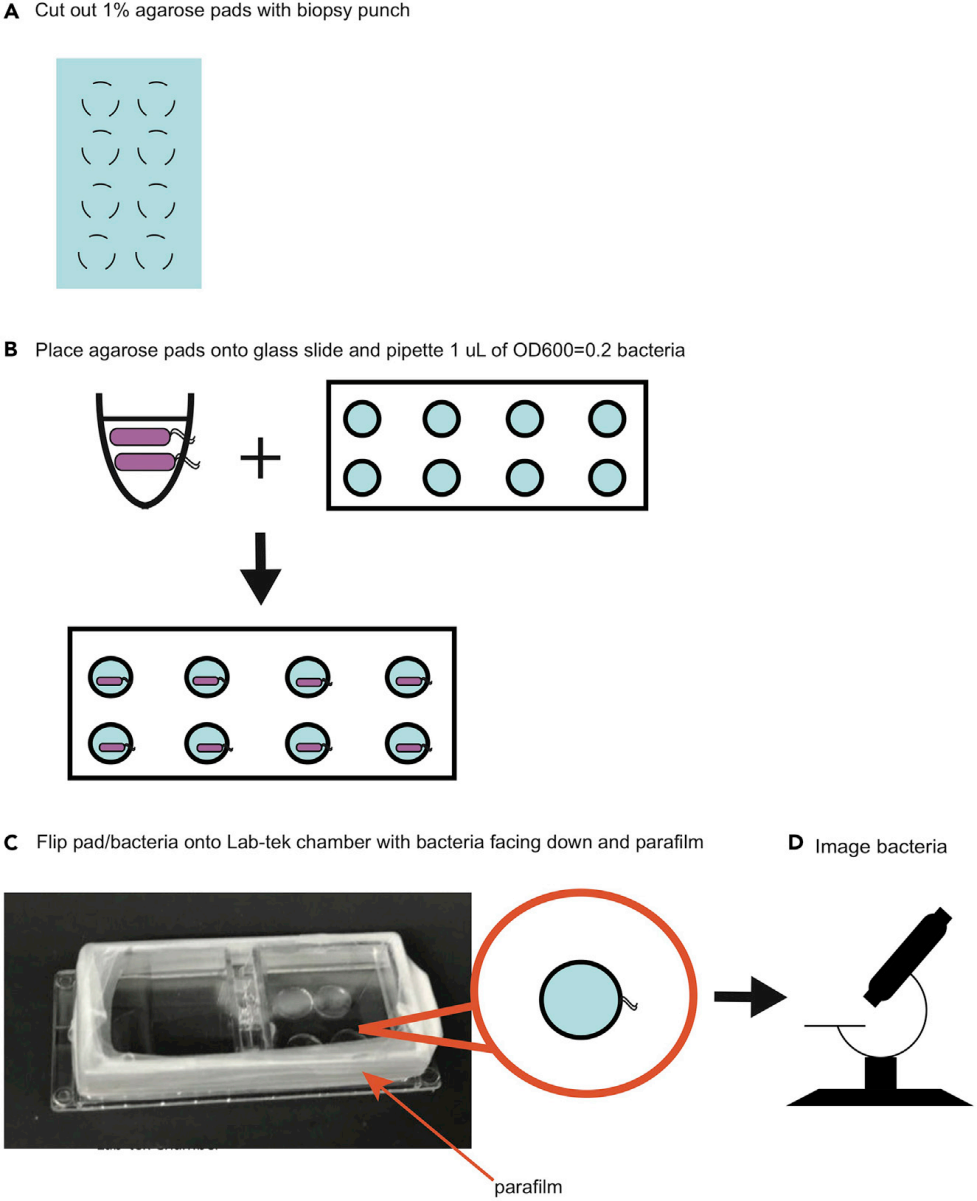
The Workflow for Generating Agarose Pads with Bacteria for Live-Cell Imaging (A) Individual agarose pads are cut from a slab of 1% agarose with a biopsy punch. (B) Pads are transferred to a glass slide, and bacteria are pipetted to the center of each pad. (C) Pads are placed, bacterial side down, on a Lab-tek chamber. The chamber is sealed with parafilm prior to imaging. (D) The agarose pads are placed on a microscope with a 37°C environmental temperature chamber for live-cell imaging.
68. Wait 5 min for bacteria to dry onto the pads.
69. Using a scalpel, transfer pads into a Labtek Chambered Coverglass (1 well, Fisher Scientific) upside-down.
70. After all pads are transferred, wrap the Labtek junction between the Labtek cover and base several times around with Parafilm, to maintain moisture (Fisher Scientific).
71. Transfer the Labtek to a microscope.
72. Follow the protocol in: Live-cell Imaging Conditions, steps 82–83. See Methods Videos S1 and S2 for example time courses of *E. coli* λ-mKate lysogens growing on an agar pad with and without NFX stimulation.

Methods Video S1. WT cI λ Phage with Late Lysis mKate Reporter Imaged on Agar Pad for 6 h at 37°C, Related to Section Live-Cell Imaging Conditions: Steps 82–83 Overlay of bacteria (phase image) and mKate phage reporter (red). Scale bar, 10 μm. Adapted from Bodner et al., 2020, Video S2.

Methods Video S2. WT cI λ Phage with Late Lysis mKate Reporter Imaged on Agar Pad for 6 h Post-norfloxacin Treatment at 37°C, Related to Section Live-Cell Imaging Conditions: Steps 82–83 Overlay of bacteria (phase image) and mKate phage reporter (red). Scale bar, 10 μm. Adapted from Bodner et al., 2020, Video S3.

### Bacterial Lysogen Infection of RAW264.7 Cells

**Timing: 2 days**

This section contains the steps involved for infecting macrophages with lysogenic and non-lysogenic bacteria and subsequently preparing the cells for imaging to observe prophage induction inside of phagocytosed *E. coli*. In our model system, the mammalian cells and bacterial cells have both been engineered to constitutively express fluorescent proteins compatible with live-cell imaging. The RAW264.7 macrophages have been engineered to contain a far red nuclear marker - the fluorescent protein miRFP670 is fused to Histone 2B. The *E. coli* strain contains a plasmid with a constitutive promoter that expresses mCerulean3. For details on creation of these strains, see Bodner et al., 2020 and the Key Resources Table.

73. One day prior to imaging prepare bacterial and mammalian cell cultures.
a. Seed RAW264.7-H2B-miRFP670 cells at 1.5 x 10^4^ cells/well on a 2 μg/mL fibronectin-coated (Sigma, F0895) 96-well glass imaging plate (Fisher Scientific, 164588) in DMEM supplemented with 10% FBS (Omega Scientific) and 2 mM L-Glutamine (Life Technologies).

***Note:*** The seeding density is determined such that the macrophages are less than 50% confluent on the day of infection.

b. Grow up from single colonies of lysogenic and non-lysogenic strains overnight (∼16 h) in LB supplemented with the appropriate antibiotic.
74. 16 h after seeding RAW264.7 cells start the infection workflow. Pre-heat a large centrifuge, with plate adapters, to 34°C.
75. Back-dilute *E. coli* (lysogenic and non-lysogenic) from those that were grown for 16 h in LB cultures, into fresh LB (without antibiotics) and grow to mid-exponential phase (OD600=0.4). Grow all *E. coli* strains at 37°C.

***Note:*** Always include a non-lysogenic strain control to monitor macrophages for efficient bacterial clearance and to have a negative control strain for the phage fluorescence reporter.

***Note:*** We optimized the strain growth phase by trying mid-exponential (OD600=0.4), freshly stationary (OD600=2.0) and saturated stationary strains (OD600=4.0). The mid-exponential phase *E. coli* are cleared the slowest by the macrophages, giving a longer time period for phage induction to occur. For other bacterial strains such as *Salmonella*, stationary phase cells may be used for the purposes of virulence factor induction.

**CRITICAL:** Always maintain RAW264.7 cells in antibiotic free DMEM prior to infection. If cells were maintained with antibiotics prior to the start of the experiment, wash the RAW264.7 cells twice with imaging media (IM), and add back 150 μL IM.

***Alternatives:*** For experiments using temperature sensitive phage, grow lysogens at 30°C.

76. Wash bacteria twice with PBS and resuspend at the appropriate concentration to achieve MOI 10 for live-cell experiments.
a. For MOI 10, since there are 15,000 macrophages/well, add 150,000 *E. coli*/well containing macrophages.
b. We make the assumption that OD600 of 1.0 = 8 x 10^8^ bacterial cells/mL. For other strains of bacteria, you may need to do colony formation unit assays to determine the relationship between OD and cell count.

***Alternatives:*** MOI can be manipulated depending on the nature of the experiment. We optimized by trying MOI 1, 5, 10 and 30. While prophage induction was observed with each MOI, 1 and 5 required imaging many more cells, and MOI 30 led to poor macrophage health.

77. To infect RAW264.7 cells, add 5 μL per well of the lysogenic or non-lysogen bacterial strain. As a control, leave at least 2 wells uninfected to monitor cell viability during the imaging time course.
78. Centrifuge plates at 200g at 34°C for 15 min, and incubate at 37°C for 1 h.

***Note:*** Since this is a long centrifugation step, we set the temperature of the centrifuge as close to 37°C as possible without overheating.

***Note:*** During incubation step, prepare the microscope settings and begin heating the environmental chamber to 37°C.

***Alternatives:*** Incubation time window is flexible; times can range from 30 min to 1 h depending on the nature of the experiment. Shorter incubations can be used to avoid missing the induction time window of temperature sensitive phages*.*

79. Wash cells two to three times with imaging media (IM).

***Note:*** When washing, make sure to pipette from a consistent corner of the well rather than the center. Pipetting into the center of the wells could disturb the cell monolayer.

80. Add back 150 μL IM supplemented with gentamicin to each well.
81. Seal the plate with an AeraSeal and immediately image the cells with the lid removed to ensure adequate gas exchange.

### Live-Cell Imaging Conditions

**Timing: 2 days**

In this final section, settings and conditions necessary for microscope preparation are outlined for both bacterial agar pad and RAW264.7 live-cell imaging.

82. For all imaging experiments, we use a Nikon Eclipse Ti fluorescence microscope, encased in an environmental chamber maintained at 37°C along with 5% CO_2_.
a. The microscope is controlled by Micro-Manager.
b. All images are acquired on an Andor Neo 5.5 sCMOS camera.
83. For imaging agar pads:
a. At least 2 images/pad are taken in every 10 min intervals with a 40×/0.95 numerical aperture objective + 1.5× tube lens with 2 × 2 binning, at 37°C for 3–5 h.
84. For imaging infected mammalian cells:
a. Images are taken at 1 h intervals with a 20×/0.75 numerical aperture objective with 2 × 2 binning for at least 18 h.
b. Image an uninfected control well in order to monitor cell health throughout the time course.
c. At least 3 wells per strain and 9 fields-of-view are imaged per well.

***Note:*** 1 h interval imaging is required for a phage induction imaging experiment to allow for the imaging of 6 strains in parallel. A tradeoff exists between the number of positions that can be imaged and the interval between time points. Shorter time intervals may be used if one is imaging fewer strains.

**CRITICAL:** Focus images using the bacterial channel to ensure the bacteria stay in focus throughout the time course.

***Alternatives:*** Imaging time intervals, objectives, and binning can be changed depending on the nature of the model experimental system used.

**Figure 11:**
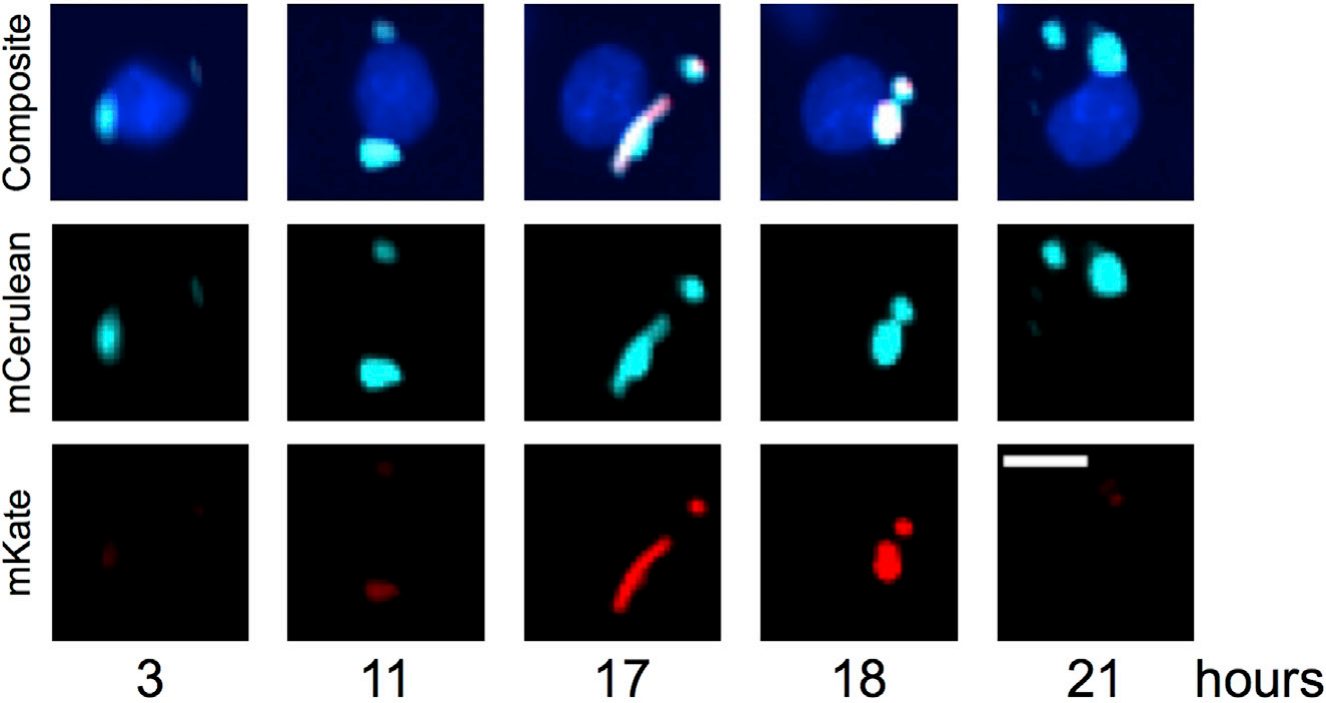
Still Images from a Time Course of a Single Tracked RAW264.7 Macrophage with Phagocytosed Bacteria that Undergo Prophage Induction and Lysis Upper panel is an overlay of nuclear marker H2B-miRFP670 (blue), bacterial marker mCerulean3 (light blue) and phage lysis marker mKate (red). Middle panel is bacterial marker only. Bottom panel is phage marker only. The scale bar, 10 μm. Adapted from Bodner et al., 2020.
a. The fluorescent markers dictate which microscope filter sets to use:
i. DIC - for cell morphology
ii. FarRed - for nuclear marker
iii. CFP - for bacterial marker
iv. TRITC - for phage marker

85. The engineered *E. coli* λ lysogens contain a constitutive mCerulean3 plasmid as a bacterial cell marker and an mKate2 fluorescent protein in the late lysis operon. RAW264.7 macrophages contain an H2B-miRFP670 nuclear marker. See Figure 11 for example images from a 21 h time course.

**CRITICAL:** To avoid phototoxicity due to imaging in the CFP channel (mCerulean3), one can use the Neutral Density Filter (ND)-60 setting to dampen the intensity of light to achieve shorter interval imaging (<30 min). The 60 refers to the average transmitted light percentage by the filter, meaning an ND-60 filter transmits 60% of the incident light from the light source.

## Expected Outcomes

In yeast-based assembly of the λ phage, with an eight-piece assembly (seven phage fragments and the YAC), screening should yield at least one out of every eight colonies that contain all junctions of the phage genome assembled correctly. PCR bands should be bright for each junction check PCR. For an example gel of the junction check PCRs, see Bodner et al., 2020, Figure S1B.

For the recombinant phage particle formation, after electroporation, there should be at least more than ten colonies on the plate. After the plaque assay with the susceptible strain, at least one cloudy plaque should form. Figure 9 shows examples of cloudy plaques for λ-mKate. After plate lysis propagation, phage titers should reach close to 10^1^^0^ pfu/mL.

After lysogen creation with recombinant phage particles, colonies should grow up in kanamycin supplemented media within 16 h. We have found that pseudo-lysogens are clear after overnight culture with some growth after two days. If lysogens are not able to grow or the plaque was not very cloudy, there is a possibility that the phage obtained a mutation during propagation. If there was a mutation in the lambda repressor, *cI*, that renders the protein temperature sensitive or inactive, then a lytic only mutant, incapable of lysogen formation, could have been generated. A PCR can be performed across the *cI* gene and then purified and sequenced to confirm that there is not a mutation.

For the lysis reporter functional validation, with λ-mKate, we observe on average that after 6 h of growth on agar pads, 7.7 × 10^−3^ *E. coli* express the lysis reporter whereas this is 1.7 × 10^−2^ with the norflaxacin treated strain. The mean phage reporter signal should be at least 1.5 times greater than background. Furthermore, the agarose pads are able to be imaged without significant shrinking for at least 6 h at 37°C, with proper parafilm wrapping of the Labtek chamber.

In an *E. coli* infection of unstimulated, RAW264.7 cells at MOI 10, we typically observe 80% of RAW cells infected with bacteria at 3 h post-infection. By 21 h post-infection, roughly 74% of infected cells fully clear the infection. By 21 h post-infection, with MG1655 *E.* coli, we typically see 20% of infected RAW264.7 cells containing at least one bacterium with prophage induction. Figure 11 shows an example infected cell with a bacterium undergoing prophage induction and lysis after 11 h post-infection. For more quantification of prophage induction throughout the time course, see Bodner et al., 2020. With proper CO_2_ supplementation to the air flow, we are able to grow the RAW264.7 cells on the microscope without significant death for at least 27 h. The cells should be dividing, even when infected with *E. coli*, while grown on the microscope. At MOI 10, in unstimulated RAW cells, we observe about 15% more nuclei at 21 h post-infection than at 2 h post-infection, indicating cell division.

## Limitations

This protocol is intended for the engineering of functional, lysogenic phages with large (>10 kb) genomes. See Table 8 for a summary of the requirements of a tripartite model system for compatibility with this protocol. For purely lytic phages, Ando et al., 2015 is applicable, and for defective lysogenic prophages incapable of forming infectious particles, the typical bacterial recombineering-based methods, such as Thomason et al., 2009, are well suitable. For translation of the yeast engineering protocol into other lysogenic phage species besides phage lambda, the attP and attB sites need to be known. If these are uncharacterized, there are bioinformatics tools to predict the location of these sites in bacterial and phage genomes. The PHAST tool (Zhou et al., 2011) can predict the location of attachment sites given a sequenced bacterial genome that contains an integrated prophage. Furthermore, there must be a DNA delivery or transformation protocol for the bacterial species of interest capable of carrying the large recombinant phage genomes. Kilcher et al., 2018 present a method for rebooting of phages in gram-positive bacteria such as *Listeria* by using a replicating, cell-wall deficient version of the strain. If the intended goal is to design a lysis reporter such as the mKate2 reporter in Bodner et al., 2020 in a different phage of interest from the lambda phage, then some further understanding of the phage lysis gene architecture and regulation would be necessary for the phage design. The most direct extension of this work would be to engineer other lambdoid phages with similar genomic architecture, as reviewed in Casjens and Hendrix, 2015. For extension into non-lambdoid phages, if the lysis operon architecture is not characterized but the genome sequence is known, then web-based prediction tools such as iVIREONS (Seguritan et al., 2012) may be used to predict the location of structural proteins, which should be located in the lysis operon. The rebooting process also assumes there exists a lysogenization protocol for the phage of interest. Stimuli to induce phage receptor expression may not be known for certain lysogenic phages of interest.

**Table 8:** Requirements to Consider When Applying This Protocol to Other Phage:Bacterium:Mammalian Cell Combinations

Step	Requirement(s)
Before You Begin - Primer Design	•The bacteriophage genome is sequenced, and the relevant bacterial attB and phage attP sites are known •The phage genome region of interest to modify (for example, the lysis operon) is well annotated
Before You Begin – Prepare PCR Products	•A lysogenization protocol exists for the bacteriophage / bacterium pair •The bacterial genomic DNA can be easily extracted and purified to be used as PCR template
Recombinant Phage Particle Formation	•The bacterial strain of interest can be made highly competent for transformation of large DNA constructs •The bacteriophage of interest can both integrate into the bacterial genome and form infectious particles •The phage receptor or mechanisms to stimulate receptor expression is known for the bacterial strain of interest, such that an efficient infection can occur to form lysogens •A colony PCR protocol exists such that the lysogenic bacterial genome can be validated
Lysis reporter Functional Validation	•A stimulus (for example, UV or DNA-damaging antibiotics) is known for prophage induction for testing the functionality of the phage lysis reporter •The doubling rate of the bacterial strain of interest is compatible with live-cell imaging
Bacterial Lysogen Infection of RAW264.7 Cells	•The bacterial strain can be transformed with a plasmid constitutively expressing a fluorescent protein marker •The mammalian cell line of interest can stably express a nuclear, fluorescent protein marker •An infection protocol exists for the bacterial/mammalian cell pair
Live-Cell Imaging Conditions	•The mammalian cell line can adhere to the imaging plate and survive on the microscope for the duration of the bacterial infection (~24 h)

The live-cell imaging protocol assumes there exists a macrophage cell line with a nuclear marker, which makes it difficult for to test prophage induction in primary cell culture. If one wants to try other mammalian cell types, there could be limitations in determining bacterial infection efficiency and optimizing the proper MOI. The environmental conditions for microscope setup (temperature, humidity, CO_2_, length of time course) may need to be fine tuned depending on cell types used. Certain light sources may contribute to high photobleaching during short time-interval imaging, making it difficult to perform at least 18 h time courses. If testing different phages of interest in macrophages, there is a chance that not all of the bacterial strains will undergo functional prophage induction inside macrophages. It may be difficult to distinguish between a negative result and under sampling (not imaging enough cells). If that is the case, instead of live-cell imaging, one can try a time course of fixing (3% formaldehyde) the infected macrophages at different time points after infection and scanning and imaging the entire plate to look for rare induction events.

## Troubleshooting

### Problem 1

PCR reactions fail for phage insert PCRs.

### Potential Solution

The genomic region may be GC rich. HF buffer can be replaced with Phusion 5× GC Buffer. Otherwise, the genomic region may have problematic secondary structures. One can titrate DMSO up to a maximal concentration of 5% of the reaction. If replacing buffers does not help, redesign the primers to target ∼50% GC content, and make sure that the first and last bases of the primers contain C or G. If no modifications to the primers or buffers help, try an alternative high-fidelity polymerase such as (Kapa HiFi Kapa Biosystems) or Primestar (Takara). Try using a polymerase with ∼10^−7^ error rate for the long 10 kb phage template PCRs.

### Problem 2

Spontaneous induction yields no plaques.

### Potential Solution

This protocol may under sample the lysogen culture. If the frequency of induction is < 10^−7^, then plating 100 μL of lysogens will yield on the order of one plaque per plate. Try plating all 3 mL of culture across 30 plates. The growth phase may affect the potential for spontaneous induction. Instead of performing the back-dilution step, try using 100 μL of a saturated stationary phase culture (OD600 > 2.0) directly to mix with plating cells. If neither of these work, the spontaneous induction frequency may be too low to detect by plaque assay, and one can perform the back-dilution step with an active inducer by supplementing the growth medium with norfloxacin or mitomycin C. Otherwise, the lack of plaque production may point to a deficiency with the engineered virus. One can take the lysogen strain and re-validate all the phage junctions through PCR (as in Yeast Engineering, step 20) and ensure that recombination did not happen during the lysogen creation step. Finally, make sure that the plating cell strains are not contaminated with phage, which would block a super-infection from taking place during the plaque assay.

### Problem 3

Yeast transformation efficiency is low.

### Potential Solution

We have found that the PEG is the reagent in the transformation mixture that most directly determines transformation efficiency. Make sure to make the PEG fresh within a day of transformation. Also, do not reuse and freeze/thaw salmon sperm aliquots. Otherwise, try to reduce the time between the final LiAc resuspension step and adding the DNA to the competent cells. Try a freshly streaked colony of yeast, and do not use plates more than several weeks old. Finally, if homemade competent cells are not efficient enough, one can try a commercial competency kit such as the Frozen-EZ Yeast Transformation II Kit (Zymo, T2001).

## Resource Availability

### Lead Contact

Further information and requests for resources and reagents should be directed to and will be fulfilled by the Lead Contact, Markus Covert (mcovert@stanford.edu).

### Materials Availability

This study did not generate new unique reagents.

### Data and Code Availability

This study did not generate any unique datasets or code.
